# 

**Published:** 1893-08-05

**Authors:** 


					*1 XAJ i-i-i-xrzyj
? PRICP TtA/nockinc W W W,TH extra nursing supplement.
^ss-^wasw pf- H jE/;
m
5th, 1893. - 1L JT* ^L/y
No. 358. Vol. XTV. Saturday,
August 5th, 1893.
[Twopence.
m

				

